# Reviewed article: Nogueira MRN, Braga HFGM, Sousa VTS, Maciel NS, Melo ESJ, Vasconcelos PF, Sousa LB. Temporal trend of HIV prevalence among persons deprived of liberty in Brazil, 2017-2023: a time series analysis. Epidemiol Serv Saude. 2025:34;e20240493.

**DOI:** 10.1590/S2237-96222025v34e20240493.a

**Published:** 2025-06-20

**Authors:** Kaciane Corrêa Mochizuke

**Affiliations:** Prefeitura Municipal de Porto Murtinho, Secretaria Municipal de Saúde

**Table:** 

Rating	Excellent	Good	Average	Below average	Poor
Interest		✓			
Quality				✓	
Originality			✓		
Overall				✓	

**Table:** 

Questionnaire	Yes	No	Not applicable
Does the manuscript contain new and significant information to justify publication?	✓		
Does the Abstract (Summary) clearly and accurately describe the content of the article?	✓		
Is the problem significant and concisely stated?	✓		
Are the methods described comprehensively?		✓	
Are the interpretations and conclusions justified by the results?		✓	
Is adequate reference made to other work in the field?	✓		

## Manuscript Structure

Length of article is: Adequate

Number of tables is: Too little

Number of figures is: Adequate

Please state any conflict(s) of interest that you have in relation to the review of this paper (state “none” if this is not applicable): None

## Comments

1) O método de análise estatística não demonstra o número da população, apenas apresenta os resultados das análises estatísticas. Quantos casos de HIV ocorreram a cada ano? Como o leitor consegue interpretar a VPA?

2) Mesmo observando o item acima o resultado precisaria ponderar a população prisional e seu crescimento anual. Seria necessário por exemplo calcular uma proporção ou taxa de HIV pela população prisional de cada ano.

3) Sugiro análise estatística adicional por terem empregado abordagem pouco adequada, para trazer maior robustez ao estudo. Afinal, da maneira exposta não deixa claro se o número de prevalência de HIV aumentou apenas porque com o passar dos anos houve um aumento no número de pessoas em privação de liberdade e essas variáveis são proporcionais, ou se de fato existem determinantes sociais de saúde que influenciam a prevalência da doença em populações do sistema carcerário.

4) No item IDH foi trazido apenas a discussão do resultado, onde está o dado? Deve trazer o resultado em uma tabela para demonstrar de maneira concisa o dado, não apenas o resultado da análise estatística

5) Na descrição da metodologia para os termos: (IDHM), Taxa de envelhecimento (T_ENV), Percentual de vulneráveis à pobreza (PPOB), Renda per capita (RDPC), Taxa de analfabetismo - 25 anos ou mais de idade (T_ANALF25M) e Renda Ajustado à Desigualdade (IDHMAD_R), não usar texto telegráfico ou tentar induzir sentido, não deve apresentar construções com uso de palavras para traduzir o sentido desejado, como consta as orientações aos autores.

6) Revise a terminologia, garantindo que estejam de acordo com os padrões científicos e que sejam compreensíveis para o público-alvo, verifique a repetição do significado de HIV e aids ao longo do texto. O termo HIV e AIDS dispensam descrição de sua sigla.

7) Verifique as regras de ortografia seguidas na construção do texto, exemplo: Vírus da Imunodeficiência Humana. O uso de maiúsculas somente deve ocorrer em casos previstos na língua portuguesa, como início de frases, cidades, países etc.

8) Sugiro buscar referências bibliográficas mais atuais, tendo em vista que o preconizado pela revista é citar pesquisas científicas relevantes, metodologicamente bem conduzidas, que foram avaliadas na íntegra pelos autores e atualizadas de até 5 anos.

